# Perspectives and Meaning of Role Transition Lived by Experienced Nurses: A Phenomenological Study

**DOI:** 10.1155/jonm/1428668

**Published:** 2025-09-26

**Authors:** Valeria Caponnetto, Francesca Fornaciari, Vittorio Masotta, Ilaria Paoli, Cristina Petrucci, Loreto Lancia, Angelo Dante

**Affiliations:** ^1^Department of Life, Health & Environmental Sciences, University of L'Aquila, Via Giuseppe Petrini, L'Aquila 67100, Italy; ^2^Hospital Facility “San Camillo Forlanini”, Circonvallazione Gianicolense, 87, Rome 00152, Italy

**Keywords:** experienced nurse, lived experience, phenomenological, role transition

## Abstract

**Background:** The role transition of experienced nurses is a complex process influenced by multiple factors that remain underexplored. Schlossberg's transition model defines three stages: “Moving In,” “Moving Through,” and “Moving Out.”

**Objectives:** The main aim was to explore the lived experiences and meanings of experienced nurses undergoing role transition. The secondary aim was to investigate main challenges faced by nurses, the perceived impact of the COVID-19 pandemic, and nurses' advice on support strategies during role transition.

**Design:** Descriptive phenomenological study.

**Methods:** In 2022, nurses who had transitioned roles within the past two years were recruited via snowball sampling. After informed consent, participants completed an online demographic questionnaire and were interviewed by phone. Interviews were audio-recorded, transcribed, and analyzed using a four-step phenomenological method: bracketing, intuition, analysis, and description. A thematic analysis was performed, and its results described following Schlossberg's transition model.

**Results:** Sixteen nurses participated (62.5% female, mean age 32.1 years, 6.6 years' experience pretransition). Four themes and nine categories emerged. Role changes were driven by personal or professional fulfillment, often tied to emotional needs or job security. Transition phases involved emotional turbulence, including anxiety, excitement, a sense of chaos, and frequent feelings of inadequacy. Support was primarily informal, coming from peers or supervisors. The whole process led to professional and personal growth, skill development, and increased self-awareness and confidence. Key challenges included unfamiliar organizational models, resistance from established teams, and emotional strain, especially when relocating. Lack of structured onboarding and the COVID-19 pandemic intensified difficulties. Participants recommended structured onboarding programs and to recognize previous experience and individual interests when assigning new roles.

**Conclusions:** Role transition of experienced nurses is both challenging and enriching. Formal support and recognition of nurses' prior experience are essential during allocation. Daily life challenges and pandemic-related issues should be considered when developing supporting strategies for transition.

## 1. Introduction

Human experience is characterized by inevitable processes of change that can unfold in various life contexts such as work, relationships, health, or other spheres. Each change manifests as a transition, a dynamic journey through the “confusing nowhere of in-betweenness” [1]. This process, whether planned or unforeseen, typically involves a period of adjustment. This period facilitates disengagement from the previous state while enabling effective preparation for the transition into a new state [2]. The transition process generally consists of three phases, which can be defined as follows: 1. “Endings”: when individuals acknowledge what they have lost. This stage is often accompanied by negative attitudes and emotions such as struggle, resistance, anger, and anxiety, which are necessary to process the loss. 2. “Neutral zone”: when individuals develop new perspectives, learn, and work the transition process. This phase is often marked by failures in new activities and negative feelings such as distress, anger, and disenchantment. 3. “New beginning”: when individuals are finally ready to overcome the transition and face the new situation. At this stage, they have usually reached good mastery of the new situation [1, 3]. Similar phases have been described in Schlossberg's transition model, which defines the phases as “Moving In,” “Moving Through,” and “Moving Out” [4]. These phases represent different aspects of an individual's experience during a transition, including the entry into a new situation, navigating through the changes, and eventually moving out or completing the transition. Based on this model, the impact of transition on individuals and their coping abilities depends on the interaction of various factors linked to four dimensions, known as the “4Ss”: situation, self, support, and strategies. “Situation” includes contextual aspects (e.g., environment, transition characteristics, related triggers, and the individual's control over transition occurrence). “Self” refers to individual characteristics (e.g., age, personal traits, and attitudes). “Support” describes social aid received. “Strategies” indicate adopted approaches and actions implemented to cope with transition [4, 5]. Strategies may contribute to modifying the situation, reframing the meaning of the event, or managing related stress [5].

In daily clinical practice, it is well known that nurses usually care for individuals experiencing significant transitions related to their illness or life events, familiarizing themselves with this phenomenon [6]. Moreover, transitions related to the work environment and career progression are particularly significant for individuals, and they are often complex and multifaced, even when experienced by nurses [6]. Nurses' role transitions are influenced by antecedents and contextual factors, involve strong and often conflicting emotions, and significantly impact nurses' abilities, autonomy, and career progression, as well as their clinical and organizational outcomes and workforce [7–12]. However, these results are mainly related to transition from nursing student to clinical nurse, which has been more widely investigated in the literature [13–17]. Fewer studies have assessed the transition of experienced registered nurses, those who have already navigated the transition from student to nurse, and who are now moving from one clinical context to another [18–21].

A considerable amount of literature on this topic focuses on specific pathways in setting change, often investigating the transition to settings with different levels of care complexity [22–27]. Although far from being exhaustive, research related to nurses' transition into new clinical areas of practice has so far highlighted that transition is a challenging event for nurses. In particular, they struggle to develop new skills and are overwhelmed by negative emotions such as anxiety, which can lead to distrust in their own skills. However, these feelings are mitigated when mastery of needed skills and roles is achieved, which may also occur through support from colleagues and formal support systems, although these are not uniform across different healthcare facilities [18].

To achieve a profound understanding of the phenomenon, several factors need to be highlighted. First, theories and conceptual frameworks of transition from student to registered nurse may not be fully applicable to experienced registered nurses. Moreover, changes in population needs in the last decades, current shortage of nurses, the heterogeneity of international policies on placement, and mobility of nurses, as well as the orientation provided to newly hired staff, should be considered [28–31]. Finally, the “pressure test” imposed by COVID-19 has prompted global health systems to adopt strategies aimed at facilitating and streamlining nurses' transitions across various settings, further underscoring the significance of specialized nursing education [32, 33].

This study was conducted to explore the lived experiences and related meanings of experienced registered nurses who have undergone role transition, employing a qualitative research design capable of providing highly detailed data. A secondary objective was to investigate the primary challenges faced by experienced registered nurses during this transition, the perceived impact of the COVID-19 pandemic on the process, and potential support strategies for nurses navigating role transition.

## 2. Methods

### 2.1. Study Design and Context

A descriptive phenomenological study was conducted, as this methodology is considered the most appropriate for capturing and analyzing the lived experiences relevant to the study aims [34–36]. This manuscript was written according to the Standards for Reporting Qualitative Research (SRQR) checklist [37].

In Italy, the transition of nurses across settings and facilities is regulated by national laws [38, 39]. Further, the healthcare system is primarily public and managed by local facilities, although private practice and facilities are also available. Nurses usually specialize and obtain advanced skills by enrolling in specific work settings or educational courses not fully acknowledged by law. Nurses' transition occurs primarily according to facility exigencies related to the relevant care standards. During setting assignment, specialization or specific skills of nurses are not necessarily considered, although some have developed regulations to ensure that nurses' skills are adequately valued.

### 2.2. Theoretical Framework

To ensure a comprehensive understanding of the phenomenon and to contextualize themes within the key moments through which nurses navigate significant changes and adaptations during their professional transition, the study results were graphically synthesized by integrating themes into Schlossberg's transition model [7]. Moreover, an overview of the emerging themes and corresponding representative quotes from participants was included in a table following the same model. The framework represents the dynamic process of role transition, encompassing three moments: 1. Moving In, where nurses adapt to the new environment, responsibilities, and expectations. 2. Moving Through, where nurses actively engage with the challenges and changes inherent in their new role, typically involving adjustment and skill acquisition. 3. Moving Out, representing the final phase of the process.

### 2.3. Participants

During the second semester of 2022, the authors contacted participants using snowball sampling. Recruitment began with two initial participants personally known to the authors (neither of whom was involved in data collection). Additional participants were progressively identified through the professional networks of those already enrolled, generating successive waves of recruitment, although the exact number of waves was not formally tracked. Nurses who had successfully transitioned from student to registered nurse, with a minimum of 2 years of work experience and who had undergone a role transition into a new clinical area of practice in the last year, were considered eligible. Considering the disagreement in the literature on what defines an experienced nurse [40–42], with some suggesting at least 1 year [43] and others proposing a range of 2 to 5 years [44], this study categorized nurses with a minimum of 2 years of work experience as experienced nurses. This threshold was chosen to exclude novice or advanced beginner nurses who might still face the challenges of early practice and to ensure that participants had already consolidated their professional role before undergoing a new transition within the last year. This approach allowed to specifically capture the phenomenon of a recent role transition, whether participants had accumulated only 2 years or many more years of professional practice.

Participants were contacted by phone to explain the nature and aims of the study. In accordance with the phenomenological study design, the sample size was not predetermined. Enrollment ended when data saturation was achieved [36].

### 2.4. Procedure and Data Collection

The study was conducted between June and August 2022, while data were analyzed between September and December 2022. Experienced registered nurses who signed informed consent were emailed an online questionnaire to collect their sociodemographic characteristics. They were then phoned to schedule an audio-recorded telephone interview. Sociodemographic data included sex, age, number of years of practice, previous work settings along with “left” and “moved in” work settings, job contract, i.e., permanent or fixed term and public or private practice, and workplace location, i.e., Italian region, categorized as Northern, Central, or Southern Italy [45].

The guiding questions were developed based on relevant literature and a focus group and are presented in Table 1. Prior to data collection, two pilot interviews were conducted to test the pertinence of the questions, identify potential issues, and refine the researcher's interviewing style. These interviews did not result in substantial modifications to the pre-established guiding questions but confirmed their appropriateness for the study aims. To encourage reflection on personal experiences, participating nurses were emailed the guiding questions 1 week before the scheduled interview. To maintain consistency, the same researcher conducted all interviews. The lead researcher, a female PhD, served as a research fellow during the study. She has been affiliated with the university for 3 years and had no prior interactions with participants. Nurses were informed that study outcomes would shape future practices and research and that the researcher had no prior exploration of the topic. No deviations from initially conceptualized methodology occurred.

### 2.5. Data Analysis

Descriptive statistics were used to summarize participants' characteristics. Interview recordings were carefully listened to and transcribed verbatim immediately after each call. In line with the phenomenological approach, participants' experiences were recoded in four specific stages: (1) bracketing, (2) intuition, (3) analysis, and (4) description [36]. Within this framework, thematic analysis was conducted manually, consistently with the phenomenological design and the relatively small sample size, which enabled close engagement with the data and enhanced reflexivity [34, 46]. First, to ensure objectivity and avoid bias, authors engaged in thorough discussions and openly shared their own opinions about the participants' experiences while temporarily suspended judgment. In the second phase, two researchers independently immersed themselves in the phenomenon of nurses' role transition by reading and re-reading the interviews and field notes to deepen understanding. In the third phase, the two researchers independently coded all transcripts, extracting the most relevant statements and categorizing them using the researchers' language. Discrepancies were discussed until consensus was reached, involving a third researcher when necessary. Finally, meanings and experiences were synthetized into themes by grouping related concepts. Quotations for key statements are included in the results (e.g., Interview 5). Triangulation was carried out by the research team to confirm findings and minimize potential bias [47]. Data analysis ensured inter-rater reliability and confirmability.

### 2.6. Ethics

This study was conducted in accordance with the Helsinki Declaration. Verbal and written informed consents were obtained from all participants: verbal consent was recorded at the beginning of each interview, and written consent was collected via signed form returned by email. Anonymity was maintained by assigning each participant a unique numerical code to replace their name during interviews and in audio recordings, which was also used in the transcripts. Audio recordings were transcribed and securely stored. Data processing followed national privacy regulations, exclusively for scientific purposes and guaranteeing nonidentification of participants [48].

In line with local and European regulations [48, 49] and considering the qualitative nature of the study, which was conducted within the institutional framework of the Master of Science in Nursing program, approval was obtained from the Board of the Master of Science in Nursing Course on 20th April, 2022.

### 2.7. Rigor and Reflexivity

Trustworthiness was ensured according to Guba's criteria [50], covering authenticity, credibility, confirmability, dependability, and transferability. The authors provided a detailed and transparent account of procedures, methods, and setting, maintaining consistent observation through deep familiarization with the phenomenon. Guiding questions were shared with participants before the interview, allowing introspection and enhancing data depth. Questions were not used as a strict framework to be followed, but rather as a guide to capture a comprehensive view of the interviewee's lived experience. One researcher conducted all interviews with a purposive sample of nurses, taking field notes that informed debriefing sessions and guided subsequent interviews. The researcher had no personal connections with interviewers, ensuring the absence of personal bias. Bracketing and triangulation were applied to ensure objectivity and focus on participants' experiences, following Husserl's phenomenological philosophy. Interviews were transcribed verbatim, guaranteeing that researchers' personal interpretations did not distort the data. Results were presented in a visual framework based on the literature, alongside a table including key quotations to ensure clarity.

## 3. Results

### 3.1. Characteristics of Participants

Data saturation was reached after consecutively contacting and interviewing 16 registered nurses. Most participants were female (*n* = 10; 62.5%), with a mean age of 32.1 years (SD = 4.8; median = 31.5, range = 27–42). On average, nurses reported 6.6 years of practice before the transition (SD = 4.6; median = 5.5, range = 2–17), and all had worked in more than one setting prior to the transition. During the investigated transition, participants left and moved to heterogeneous settings, including COVID-19 areas, as detailed in Table 2. Although all transitions were voluntary, participants did not have the opportunity to choose the new work setting. Nurses mainly left permanent contracts in private practice (*n* = 8; 50.0%) or, less frequently, permanent contracts in public practice (*n* = 4; 25.0%). Conversely, “moving-to” job contracts mainly included permanent contracts in public practice (*n* = 9; 56.3%) or, less often, permanent contracts in private practice (*n* = 4; 25.0%). Ten nurses (62.5%) left Central Italy, and a similar number (*n* = 11; 68.8%) moved to the same geographical area. Transitions related to Northern and Southern Italy interested a few participants (Table 2).

### 3.2. Interviews

**Figure 1:**
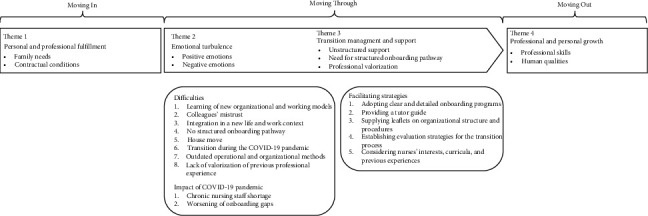
Study results embedded within Schlossberg's transition model.

On average, the interviews lasted 15.3 (SD = 4.5) minutes, with a range of 8.2–23.2 min.  Figure 1 depicts the results of the interviews analysis according to Schlossberg's transition model, highlighting 4 main themes and 9 descriptive categories related to nurses' experiences. Additionally, the main difficulties faced by nurses during transition and the impact of the COVID-19 pandemic were investigated and described. Results in detail are described below, and a summary with the most representative quotations is provided in Table 3.

### 3.3. Moving In Phase

In the first phase, it emerged that nurses, when facing the new environment and responsibilities, were guided by their personal motivations. During this stage, nurses were prompted to align their personal aspirations with the demands of their new roles, emphasizing the significant role of personal motivations in the process of role transition.

#### 3.3.1. Theme 1. Personal and Professional Fulfillment

Nurses' motivation to move was informed by finding fulfillment in their personal or professional lives. In particular, most of them were motivated to undergo role transition because of family-related issues.

*“[…] the motivation was to live close to my original family.”* (Interview 16)

*“I decided to move […] to get closer to my family.”* (Interview 2)

Others decided to move due to improved job conditions, especially with regard to the contract.

*“I moved for the type of contract. I got a permanent job in public practice […].”* (Interview 7)

### 3.4. Moving Through Phase

In the second phase, nurses actively navigated challenges in their new roles, encompassing emotional adjustments and skill acquisition. This phase revealed emotional turbulence, with nurses experiencing a range of feelings. During this stage, nurses emphasized the importance of structured onboarding, underlining its role in facilitating a smoother transition, boosting confidence, and fostering professional development.

#### 3.4.1. Theme 2. Emotional Turbulence During Transition

Transition was described as an intense experience, characterized by conflicting emotions. These emotions could be positive, such as gladness and enthusiasm for the new job, but also negative, such as anxiety, worry, and fear.

*“I was worried because I was aware of the situation I was leaving behind, but I had no idea what I would encounter in my new surroundings. However, I was still happy because it meant that I would spend less time traveling to be with my family […]. Therefore, happiness was the prevailing emotion, although it was naturally accompanied by some worry.”* (Interview 2)

*“The strongest emotion I felt was a great chaos feeling.”* (Interview 9)

Some nurses also had feelings of inadequacy, uncertainty, and disorientation.

*“I remember very well that sense of not feeling up to experienced nurses.”* (Interview 4)

#### 3.4.2. Theme 3. Transition Management and Support

All nurses reported undergoing an unstructured onboarding program for new hires during the transition. They mainly received informal support from fellow nurses already established in the new setting or other colleagues who were undergoing role transition as well. Others received support from their clinical management leads, such as charge nurses or nursing directors.

*“I received support only from colleagues who lived the same experience as me.”* (Interview 8)

*“[…] charge nurse and new colleagues supported [me] to integrate in the new working environment during the first weeks. Their support was mostly emotional rather than practical. The charge nurse gave me an overview of the general structure of the new organization […].”* (Interview 5)

Participants also emphasized their need for a structured onboarding process, which was considered beneficial in reducing the time required to achieve autonomy. Nurses recognized the potential of an onboarding pathway in boosting their confidence and awareness, as well as ensuring their professional development.

*“[…] It is different to have a nurse who received a training for over six months to manage dialysis patients than to have a nurse who was trained in a rush.”* (Interview 11)

*“If there had been [a structured onboarding program], I would have spent less time to integrate and I would have been much more independent as early as the second day.”* (Interview 10)

*“Knowing the whole contest from the beginning certainly makes you more aware and confident.”* (Interview 14)

### 3.5. Moving Out Phase

The “Moving Out” phase represents the conclusion of the role transition process. Participants reflected on their experience, evaluating achievements and challenges. Notably, it embodied both professional and personal growth, as nurses acquired new skills while enhancing their human qualities.

#### 3.5.1. Theme 4. Professional and Personal Growth

Participants perceived the transition as an opportunity for both professional and personal growth. They primarily reported professional growth associated with the acquisition of new skills.

*“Getting in touch with new realities led to greater awareness and security, even at a professional level.”* (Interview 14)

Furthermore, this professional growth is intertwined with the enhancement of their human qualities.

*“Definitely both from a professional and human point of view. This experience allowed me to mature as a person, as a man, and as a nurse.”* (Interview 15)

### 3.6. Difficulties Faced During Nursing Role Transition

Participants described several difficulties during the “Moving Through” phase of their transition. These were either closely related to the work environment, e.g., the organizational model, or to their emotions, their attitudes, or the organization of their daily lives.

The most described difficulty was having to learn new organizational and work models.

*“[…] I had to start all over from scratch.”* (Interview 15)

*“The main difficulty was gaining confidence with a different work model […].”* (Interview 1)

In this context, some nurses also dealt with mistrust and resistance from colleagues before they were integrated into the new work environment.

*“I remember the first day. I cried so hard because I found myself in an already established, very cohesive group, which for the first few weeks resisted letting me be included […].”* (Interview 2)

*“The main difficulty was staying in a place I didn't know and very far from home. Without reference points […].”* (Interview 15)

*“Certainly, meeting new colleagues [was a challenge], you know, approaching people you don't know. In addition to changing workplaces and, therefore, work types, you also have people next to you that you don't know […].”* (Interview 7)

Once again, the absence of a structured onboarding program was considered an issue.

*“Fortunately, I had previous experience that allowed me to be immediately productive. I didn't have any tutoring […].”* (Interview 6)

To a lesser degree, changing accommodation was seen as a complicating factor.

*“[…] I had to move to a new house very far from home and this was very difficult, hard, and stressful.”* (Interview 16)

Some nurses had difficulties managing the clinical conditions of COVID-19 patients in a new environment.

*“[…] we were all newly hired nurses, and we had to face something new that nobody knew [about].”* (Interview 8)

Outdated operational and organizational methods in the new work setting made transitioning more difficult.

*“Professionally, I got into trouble when I realized that in the new work environment, they adopted outdated methods compared to my previous work setting […].”* (Interview 16)

Showing no recognition for skill level and previous experiences in the new setting was perceived as another challenge.

*“My previous professional experience was not considered at all […].”* (Interview 6)

### 3.7. Impact of COVID-19 Pandemic

Participants highlighted critical issues regarding personnel transition management and chronic staff shortage in healthcare facilities due to the COVID-19 pandemic. In particular, they perceived the absence of onboarding pathways as potentially harmful for patients.

*“[…] we are even more understaffed, and it is even impossible to have proper training and mentoring because of the lack of personnel. Those who are available are assigned to COVID-19 wards or emergency departments, which are struggling.”* (Interview 11)

*“[…] nurses were hastily and superficially placed in wards during that period, at high risks to patients.”* (Interview 14)

### 3.8. Support Strategies for Nursing Role Transition

Most participants recommended implementing clear and detailed onboarding programs with clearly defined objectives and methods. Additionally, they proposed providing access to a mentor for the program and distributing leaflets outlining the organization's structure and procedures. Finally, the evaluation of the placement process was deemed advisable.

*“I think that a structured onboarding program with practical and specific aims is needed […].”* (Interview 4)

*“The newly hired nurse should be assigned a tutor.”* (Interview 3)

*“[…] it would have been useful to have information leaflets that the human resources office could provide when [new hires] sign the hiring contract and then more specific information leaflets on the assigned ward.”* (Interview 2)

*“Moreover, I think that initial, intermediate and final assessments are also useful to understand if the nurse is suitable [for the role] and well placed.”* (Interview 16)

An interesting aspect raised by nurses was the suggestion for healthcare facilities to appreciate and value nurses' interests, curricula, and previous experiences.

*“A good strategy could be performing interviews [of newly hired nurses] […] in order to understand our areas of interest and attitude and assigning us to specific settings where we would have more motivation. […] a ward is a place where one can feel confined, and after a while, we often need to change [settings], not only for professional reasons but also for personal motivations.”* (Interview 5)

*“[…] it is important to value and treasure professional experience because an already trained nurse means reduced training costs.”* (Interview 16)

## 4. Discussion

The aim of this study was to investigate the lived experiences and meanings of nurses undergoing role transition, focusing on the challenges they face, the impact of the COVID-19 pandemic, and possible support strategies. Regarding participants' characteristics, sex distribution in the sample closely mirrored the national figure, while participants were, on average, younger than the reference population [51, 52]. Due to snowball sampling, participants showed considerable variability in contract types, locations, years of practice, and expertise. This heterogeneity enriched the data and reduced the risk of excessive homogeneity in participants' narratives. At the same time, it made it more difficult to delineate highly specific patterns within particular subgroups, which may partly constrain the transferability of findings to narrowly defined populations. Nevertheless, the emergence of recurring themes across such a diverse sample reinforces the robustness of the results and points to their relevance for younger nurses with moderate experience (mean of 6.6 years), often transitioning from the private to the public sector and from less complex to more complex clinical settings.

Regarding the transition experience, the lived experiences of participants during the role transition process were surprisingly similar. In the “moving-in” phase, registered nurses were primarily motivated by personal and family-related factors, although the enhancement of job conditions was also mentioned. This finding suggests that role transition among experienced registered nurses, who were typically younger than the national reference population, was driven by the desire to strike a balance between personal and professional life. This highlights the significant impact that a career change can have on nurses' overall life satisfaction [53]. It also indicates that young registered nurses pursue job changes that align more closely with their skills and interests [41, 54]. For these reasons, it is crucial that healthcare managers consider the social contexts and ambitions of nurses when allocating human resources. Doing so can help create a healthy and stable work environment that fulfills nurses' needs and aspirations, promotes autonomy and job satisfaction, and positively reflects on organizational and patient outcomes [55].

Evidence from international research highlights the critical importance of structured and personalized onboarding programs designed to support nurses undergoing role transition, whether newly graduated or experienced, to enhance their knowledge, critical thinking, and job satisfaction [56–58]. Structured onboarding initiatives often include on-the-job training, mentoring, and ongoing evaluation, which help nurses adapt more quickly and with less stress to their new role [57, 58]. Mentorship programs have been widely adopted across healthcare systems to facilitate integration and professional development, particularly for nurses transitioning across specialties or for internationally recruited staff [59–61]. Similarly, structured preceptorship programs have been shown to enhance confidence, promote integration, and improve retention, thereby supporting nurses during critical transitions [62]. These practices foster a sense of belonging and professional growth, which are recognized as key factors in reducing turnover and improving job satisfaction. In the Italian context, where onboarding and mentorship practices remain heterogeneous and sometimes fragmented, formalizing and expanding comprehensive frameworks tailored to nurses' transitions could represent a valuable strategy to strengthen workforce stability.

Transition management also proved to be a challenging process, as it evoked conflicting emotions in nurses regardless of their prior experience. Although experiencing both positive and negative feelings is a physiological phenomenon during transition [1, 3], negative emotions appeared more frequent and should be of particular concern for healthcare facilities, since they can negatively impact nurses' well-being and the quality of care. These feelings were often intense, impacting nurses' daily life. They stemmed from a diverse range of experiences, such as adapting to unfamiliar situations, meeting new colleagues, the organization's stance toward new hires, and most significantly, the sense of inadequacy due to the absence of a structured onboarding pathway.

Since negative feelings and challenges can often be overcome through the mastery of necessary skills and roles, the implementation of onboarding programs and support strategies, including preceptorship, is crucial regardless of nurses' prior experience or expertise [18, 63, 64]. In addition to highlighting nurses' abilities to stakeholders, such programs, according to the interviewed nurses, should include essential features such as recognition of previous experiences and education, diversified orientation methods, including personal tutoring, and evaluation of program outcomes. Notably, these features largely overlap with those reported in the literature, thereby reinforcing their relevance as core components of effective onboarding [57, 58]. Recognizing previous experience and educational levels, and employing specific educational methods, may enhance the ability to make clinical decisions in unfamiliar environments, thereby reducing the observation period and facilitating a gradual increase in independence and satisfaction with the new role [64, 65].

Regarding other difficulties encountered during transition, healthcare facilities should adopt updated standards of organizational and clinical management, in addition to providing new hires with adequate logistic and social support. Careful consideration of new hires' well-being is essential to optimize their placement and, in turn, enhance organizational and clinical outcomes. The COVID-19 pandemic further underscored the urgency of addressing these issues [32, 33], highlighting long-standing management challenges in Italy, such as nursing shortage and understaffing, which now require urgent attention [66, 67].

Finally, personal and professional growth resulting from role transition suggests that healthcare facilities should allow nurses to experience different settings, thereby fostering the development of more skilled and proactive professionals.

To the authors' knowledge, this is the first Italian study investigating the life stories of experienced registered nurses who underwent a role transition process. The study's results provide valuable insights for healthcare facility managers, shedding light on the role transition of nurses. These findings have the potential to inform strategies aimed at supporting nurses during their transitions, ultimately enhancing their career development and work-related well-being. Furthermore, they may contribute to improved patient care and organizational outcomes. Nevertheless, it is important to contextualize the study within the small sample size, the qualitative methodology employed, and the specific study context [68]. These factors constrain the generalizability of findings and emphasize the need to acknowledge that the results may not apply universally. Moreover, since recruitment was conducted through snowball sampling, the exact number of referral waves was not formally tracked. While this represents a limitation in terms of potential sampling bias, the risk of homogeneity was mitigated by the diversity of participants' backgrounds and by the decision to discontinue recruitment once data saturation was reached. Although the average interview length was relatively short, this was balanced by providing participants with the guiding questions in advance, which allowed them to reflect on their experiences beforehand and ensured focused, in-depth responses within the available time. Even if caution is needed in transferring the findings beyond the study setting, they may be particularly applicable to health systems similar to the Italian context, i.e., public systems characterized by variability in contractual arrangements, a persistent nursing shortage, and limited or heterogeneous onboarding practices. Within such contexts, the results may be most relevant for nurses with characteristics similar to those of our participants (younger, moderately experienced, and transitioning toward more complex clinical settings, often from the private to the public sector). In contrast, applicability may be more limited in health systems with highly formalized transition pathways or mandatory specialization requirements. To address these limitations, future research should combine quantitative methods with qualitative insights to provide a more comprehensive view of role transitions. Additionally, evaluating the effectiveness of various onboarding programs could improve transition experiences, while investigating role transitions in different nursing specialties and across countries may help identify best practices and specific challenges.

## 5. Conclusions

This study found that experienced nurses are primarily motivated to transition to new roles from one facility to another by their desire for work–life balance and improved contractual conditions. During role transition, nurses often encounter challenges and conflicting emotions. Negative emotions highlight the need for structured onboarding programs for new hires. An ideal transition process should consider nurses' skills, ambitions, and previous experiences. It should incorporate multifaced training methods, including opportunity for personal tutorship, and should be evaluated longitudinally to ensure that the desired objectives are being met. Role transition offers opportunities for personal and professional growth. In this regard, healthcare facilities should allow nurses, especially new hires, to experience different settings, in order to improve their abilities and self-confidence. Healthcare facilities are recommended to adopt up to date standards in organizational and clinical management, while also providing new hires with comprehensive logistical and social support.

## Figures and Tables

**Table 1:** Guiding interview questions.

*Questions related to the primary aim:*
1. What were the motivations that led you to transfer to a new healthcare facility?
2. What did you feel during the role transition?
3. Who supported you during the role transition process?
4. What were the positive effects that characterized this experience?
5. What did you need during transition? Was there a structured transition program? What effects did it have on your experience?
6. What facilitating strategies can be implemented to improve the transition of a nurse to a new work environment?

*Questions related to the secondary aim:*
1. What were main difficulties that characterized this experience?
2. How has the COVID-19 pandemic influenced the role transition process?

**Table 2:** Characteristics of participants (*n* = 16).

Par	Sex	Age	Years of practice	Work setting		Contract (sector)		Italian geographical area	
				Left	New	Left	New	Left	New
1	M	34	9	Medicine	Emergency	Permanent (private)	Permanent (public)	North	Center
2	F	30	7	Intensive care	Emergency medicine	Permanent (private)	Permanent (public)	North	North
3	M	27	2	Surgery	Neurology and stroke	Permanent (private)	Permanent (public)	Center	South
4	M	36	3	Medicine	Intensive care	Self-employed	Fixed term (public)	Center	Center
5	F	31	7	Intensive care	Basic health district	Permanent (private)	Permanent (public)	South	South
6	F	32	8	Multispecialist	Oncology day hospital	Permanent (public)	Permanent (private)	Center	Center
7	F	32	10	Pediatric intensive care	COVID intensive care	Permanent (public)	Permanent (private)	Center	Center
8	F	27	2	Community hospital	COVID medicine-emergency	Fixed term (public)	Permanent (public)	Center	Center
9	F	40	2	Orthopedic	Emergency	Permanent (private)	Permanent (public)	Center	Center
10	M	38	17	Hemodynamic	COVID subintensive care	Permanent (private)	Permanent (public)	Center	Center
11	F	27	5	Nephrology	Dialysis	Fixed term (public)	Fixed term (public)	Center	Center
12	M	32	3	Outpatient clinic	Medicine	Fixed term (public)	Fixed term (public)	South	North
13	F	28	4	Oncology	COVID medicine	Permanent (private)	Permanent (public)	North	Center
14	F	42	16	Multispecialist private regimen	Medicine	Permanent (public)	Permanent (private)	Center	Center
15	M	27	4	Surgery	Mental health center H24	Permanent (public)	Permanent (private)	Center	North
16	F	31	6	Pediatric intensive care	Cardiology	Permanent (private)	Permanent (public)	North	Center

*Note:* Par = participant; F = female; M = male; H24 = 24 h active service.

**Table 3:** Overview of the emerging themes and corresponding representative quotes from participants.

Phase	Theme	Descriptive categories	Most representative quotations
Moving In	Personal and professional fulfillment	Family needs	*“I decided to move […] to get closer to my family.”* (Interview 2)
		Contractual conditions	*“I moved for the type of contract.”* (Interview 7)

Moving Through	Emotional turbulence	Positive emotions	*“[…] Therefore, happiness was the prevailing emotion, although it was naturally accompanied by some worry.”* (Interview 2)
		Negative emotions	*“The strongest emotion I felt was a great chaos feeling.”* (Interview 9)
	Transition management and support	Unstructured support Need for structured onboarding pathway	*“[…] It is different to have a nurse who received a training for over 6 months to manage dialysis patients than to have a nurse who was trained in a rush.”* (Interview 11)
		Professional valorization	*“Knowing the whole contest from the beginning certainly makes you more aware and confident.”* (Interview 14)
	Difficulties	Learning of new organizational and working models	*“The main difficulty was gaining confidence with a different work model […].”* (Interview 1)
		Colleagues' mistrust	*“I remember the first day. I cried so hard because I found myself in an already established, very cohesive group, which for the first few weeks resisted letting me be included […].”* (Interview 2)
		Integration in a new life and work context	*“The main difficulty was staying in a place I didn't know and very far from home. Without reference points […].”* (Interview 15)
		No structured onboarding pathway	*“[…] I didn't have any tutoring […].”* (Interview 6)
		House move	*“[…] I had to move to a new house very far from home and this was very difficult, hard, and stressful.”* (Interview 16)
		Transition during the COVID-19 pandemic	*“[…] we were all newly hired nurses, and we had to face something new that nobody knew [about].”* (Interview 8)
		Outdated operational and organizational methods	*“Professionally, I got into trouble when I realized that in the new work environment, they adopted outdated methods compared to my previous work setting […].”* (Interview 16)
		Lack of valorization of previous professional experience	*“My previous professional experience was not considered at all […].”* (Interview 6)
	Impact of COVID-19 pandemic	Chronic nursing staff shortage	*“[…] we are even more understaffed, and it is even impossible to have proper training and mentoring because of the lack of personnel. Those who are available are assigned to COVID-19 wards or emergency departments, which are struggling.”* (Interview 11)
		Worsening of onboarding gaps	*“[…] nurses were hastily and superficially placed in wards during that period, at high risks to patients.”* (Interview 14)
	Facilitating strategies	Adopting clear and detailed onboarding programs	*“I think that a structured onboarding program with practical and specific aims is needed […].”* (Interview 4)
		Providing a tutor guide	*“The newly hired nurse should be assigned a tutor.”* (Interview 3)
		Supplying leaflets on organizational structure and procedures	*“[…] it would have been useful to have information leaflets that the human resources office could provide when [new hires] sign the hiring contract and then more specific information leaflets on the assigned ward.”* (Interview 2)
		Establishing evaluation strategies for the transition process	*“Moreover, I think that initial, intermediate and final assessments are also useful to understand if the nurse is suitable [for the role] and well placed.”* (Interview 16)
		Considering nurses' interests, curricula, and previous experiences	*“[…] it is important to value and treasure professional experience because an already trained nurse means reduced training costs.”* (Interview 16)

Moving Out	Professional and personal growth	Professional skills	*“Getting in touch with new realities led to greater awareness and security, even at a professional level.”* (Interview 14)
		Human qualities	*“Definitely both from a professional and human point of view. This experience allowed me to mature as a person, as a man, and as a nurse.”* (Interview 15)

## Data Availability

The data that support the findings of this study are available from the corresponding author upon reasonable request.
